# Public Health Perspectives on Integrating *Artemisia annua* Tea for Uncomplicated Malaria Treatment: A Cross-Sectional Study of Perceptions and Acceptability Among Healthcare Workers in Kalima District, Maniema, DRC

**DOI:** 10.3390/tropicalmed11040105

**Published:** 2026-04-17

**Authors:** Jérôme Munyangi wa Nkola, Pierre Akilimali Zalagile, Hendrick Lukuke Mbutshu, Spartacus Kabala Munyemo, Imani Ramazani Bin Eradi, Alioune Camara

**Affiliations:** 1 PhD Program in Public Health, Doctoral School in Life Sciences, Health and Environment, Faculty of Health Sciences and Techniques, Department of Medical Sciences, Gamal Abdel Nasser University of Conakry, Conakry BP 1147, Guinea; 2 Faculty of Medicine, University of Kolwezi, Kolwezi BP 453147, Democratic Republic of the Congo; 3 Department of Nutrition, Kinshasa School of Public Health, University of Kinshasa, Kinshasa BP 11850, Democratic Republic of the Congo; pierretulanefp@gmail.com; 4 Patrick Kayembe Research Center, Kinshasa School of Public Health, University of Kinshasa, Kinshasa BP 11850, Democratic Republic of the Congo; 5 School of Public Health, University of Lubumbashi, Lubumbashi BP 1825, Democratic Republic of the Congo; 6 Academic General Secretariat, University of Kindu, Kindu BP 122, Maniema, Democratic Republic of the Congo; 7 Nursing Sciences Research, Laboratory of Education and Health Practices, UFR SMBH, University Paris, Sorbonne Paris Cité, F-93017 Bobigny, France; imaniramazani@gmail.com; 8 Nursing Sciences Section, Higher Institute of Medical Techniques of Kindu, Kindu BP 304, Democratic Republic of the Congo; 9 Department of Public Health-Epidemiology, Faculty of Sciences and Health Techniques, Gamal Abdel Nasser University, Conakry BP 1147, Guinea; aliounec@gmail.com; 10 National Malaria Control Programme, Conakry BP 6339, Guinea

**Keywords:** *Artemisia annua*, malaria, Democratic Republic of the Congo, healthcare providers, clinical acceptability, antimalarial drug resistance, *PfKelch13* mutations, traditional medicine integration, Maniema province, medical pluralism, genomic surveillance

## Abstract

**Background:** The Democratic Republic of the Congo accounts for approximately 12–13% of the global malaria burden. While international guidelines oppose the use of *Artemisia annua* infusions due to risks of sub-therapeutic dosing and resistance selection, the plant remains widely used in resource-limited regions. This study evaluates the clinical acceptability and perceptions of healthcare providers regarding the integration of *Artemisia annua* tea into formal malaria control in the Maniema province. **Methods:** A cross-sectional survey was conducted among **337 healthcare professionals** in the Kalima health district using the KoboCollect digital platform. Multivariate logistic regression was employed to identify the primary socio-professional determinants of clinical acceptability. **Results:** The overall clinical acceptability of *Artemisia annua* integration **was 81.0%,** with **82.8%** of providers perceiving the preparation as effective. Rural residency was the strongest predictor of adherence **(AOR = 6.847; *p* = 0.003),** reflecting a pragmatic response to frequent ACT stockouts and high treatment costs. Despite high acceptability, **49.0%** of providers identified the lack of clinical evidence as a major barrier, and 91.4% demanded formal training on standardized dosage and biological mechanisms. **Conclusions:** A significant “policy–practice gap” exists between international guidelines and field realities in the DRC. Healthcare providers demonstrate high readiness for integration but emphasize the absolute necessity of galenic standardization to mitigate resistance risks. To address these concerns, a complementary genomic investigation is currently underway in the same study area, comparing *PfKelch13* mutation prevalence among *Artemisia* tea users versus ACT-treated patients. This molecular surveillance will provide essential evidence to define safety parameters for future phytopharmaceutical integration.

## 1. Introduction

### 1.1. Global and Regional Malaria Burden

Malaria remains a critical global health emergency, with the World Health Organization estimating 263 million cases and 597,000 deaths in 2023 [1]. The Democratic Republic of the Congo is central to this crisis as a ‘High Burden High Impact’ country, accounting for approximately **12.4% of the global malaria burden** [2]. With its large population, the scale of the tragedy is immense, with a high number of malaria cases annually [1]. While WHO modeling assumes a case fatality rate of approximately **1:400**, a significant discrepancy exists between estimated and reported mortality: of the **67,700 estimated deaths**, only **21,300** are officially reported [2]. This gap suggests that poor access to standardized medicines likely leaves the DRC in a more precarious condition than surveillance data indicate.

### 1.2. The Emergence of Artemisinin Partial Resistance in Africa

The efficacy of ACTs is currently threatened by the emergence of *Plasmodium falciparum* kelch 13 mutations, which mediate partial resistance to artemisinin [3,4]. Historically confined to Southeast Asia, validated PfK13 mutations such as R561H and A675V have recently been documented in Rwanda, Uganda, and Tanzania [5,6]. In Rwanda, the R561H mutation has expanded locally, reaching a prevalence of nearly 20% in some regions [5]. This evolving resistance landscape necessitates urgent vigilance in the use of all artemisinin-based treatments [7,8].

### 1.3. The Artemisia annua Controversy: Standardization vs. Synergy

In response to chronic pharmaceutical supply constraints and frequent ACT stockouts in resource-limited settings like the DRC [9], whole-plant *Artemisia annua* infusions have gained significant traction as a self-reliant treatment in developing nations [10]. Pharmacological studies substantiate the hypothesis that the plant’s complex matrix—rich in hundreds of secondary metabolites, including flavonoids (e.g., artemetin, casticin), terpenes (e.g., camphene, germacrene D), and phenolic acids [11], exerts a synergistic effect with artemisinin [12]. This enhances bioavailability via faster absorption and higher plasma concentrations from herbal teas versus pure capsules [12], while inhibiting parasite efflux pumps and defense mechanisms [13].

However, the WHO maintains strict reservations against non-pharmaceutical preparations due to absent galenic standardization, with variable artemisinin concentrations in herbal teas risking sub-therapeutic dosing—a primary driver of resistant parasite strains [3,14]. Conversely, robust evidence from experimental models demonstrates that dried whole-plant *Artemisia annua* slows malaria drug resistance evolution and overcomes established artemisinin resistance, owing to its multi-target phytochemical profile [15].

### 1.4. Healthcare Providers as Gatekeepers of Health System Resilience

The formal integration of traditional herbal remedies into primary healthcare systems depends fundamentally on the perceptions and acceptance of healthcare providers [16,17]. In countries like Ghana [17] and the DRC, healthcare workers are the frontline decision-makers whose clinical attitudes influence patient adherence and the safety of therapeutic practices [18]. While some providers view integrated phytotherapy as a sustainable response to conventional drug stockouts [9], others express concern over the lack of official clinical guidelines and scientific evidence [19]. Evaluating the perceptions of these professionals is therefore essential to developing a robust regulatory framework that aligns local clinical realities with international safety standards [20]. This study specifically assesses the acceptability of Artemisia annua tea among 337 healthcare workers in the Kalima district of the DRC to inform future public health policies.

### 1.5. Study Rationale and Objectives

This research seeks to bridge the gap between traditional practices and evidence-based medicine by exploring healthcare workers’ perceptions in the Kalima district, Maniema, DRC. By identifying the professional determinants of acceptability, this study contributes to the development of informed public health strategies in regions where conventional drug supplies are frequently interrupted [9,21]. Specifically, we aimed to determine the extent of willingness to integrate Artemisia annua tea into uncomplicated malaria treatment and identify the socio-professional factors associated with this choice. This investigation is particularly timely given the documented emergence of artemisinin resistance markers in East Africa and the potential for sub-therapeutic dosing in unstandardized herbal preparations to drive further selection of resistant strains [3], underscoring the urgency for evidence-informed integration [19]. Such integration is crucial given that a significant portion of the African population, particularly in rural areas, relies on traditional medicine due to accessibility and affordability, a trend exacerbated by events like the COVID-19 pandemic [14,22].

## 2. Literature Review

### 2.1. Molecular Markers of Plasmodium falciparum Resistance

Malaria continues to pose a formidable challenge to global health, with the 2024 World Malaria Report estimating 263 million cases and 597,000 deaths for 2023 [1]. The Democratic Republic of the Congo remains a high-burden focal point, being one of five African countries sharing 52% of the global case burden [1]. The cornerstone of malaria control, ACTs, is under pressure from mutations in the *P. falciparum* kelch13 protein. Validated mutations such as **R561H, P441L, C469Y, and A675V** have been identified as drivers of delayed parasite clearance [4,23]. In Rwanda, the **R561H** mutation has shown rapid local expansion, reaching a prevalence of nearly **20%** in certain regions [5,6]. Those mutations primarily increase selection pressure on partner drugs, leading to ACT failure rather than immediate clinical failure, which underscores the need for continuous genomic surveillance [3,5].

### 2.2. Phytochemical Synergy: The pACT Hypothesis vs. Isolated Compounds

The scientific interest in whole-plant *Artemisia annua* often termed plant Artemisinin Combination Therapy (pACT) stems from its complex biochemical matrix comprising hundreds of secondary metabolites that exert synergistic effects superior to isolated artemisinin [12]. The plant contains these bioactive compounds, including:

- Flavonoids, such as artemetin and casticin, which provide antioxidant properties and enhance antimalarial synergism [11,24];
- Terpenes, including camphene and germacrene D, contributing to the essential oil profile [11];
- Phenolic acids, which bolster overall antioxidant and antiparasitic activity [11].

#### Mechanisms of Synergy

Pharmacokinetic evidence demonstrates these compounds act synergistically with artemisinin, enhancing bioavailability through faster absorption (peak plasma concentrations reached in 30 min versus over 2 h for capsules) and solubilizing effects from other constituents, despite lower artemisinin doses in teas [12]. Moreover, metabolites such as dehydoartemisinin, dihydroartemisinic acid, and arteannuin B inhibit P-glycoprotein efflux pumps—similar to verapamil—reducing artemisinin translocation and boosting in vivo antimalarial activity against resistant strains [13]. This multi-target profile not only overcomes established artemisinin resistance but also slows its evolution in experimental models [13,15].

### 2.3. Standardization Challenges and Selection Pressure

Despite hypothesized synergistic advantages, the lack of galenic standardization remains a major concern. Artemisinin concentrations in infusions fluctuate based on plant chemotype, water temperature, and infusion time. Inconsistent dosing risks exposure to artemisinin levels that are inadequate for total parasite eradication but sufficient to impose selective pressure favoring resistant strains [3,8]. This “uncontrolled drug pressure” could expedite the obsolescence of existing artemisinin-based therapies [3,14]. However, experimental evidence counters this by showing that dried whole-plant *Artemisia annua* slows malaria drug resistance evolution and overcomes established artemisinin resistance due to its multi-target phytochemical profile [15].

### 2.4. Integrating Traditional Medicine into Formal Regulatory Frameworks

The integration of herbal products like *Artemisia annua* into primary healthcare systems requires a balance between local therapeutic resilience and international safety standards [25,26]. In many endemic regions, traditional medicine is the first line of defense due to its affordability and accessibility, particularly when conventional drug supplies are interrupted [10,27]. However, transitioning from “informal usage” to “clinical integration” requires a robust national regulatory framework that ensures product quality, standardized dosing, and rigorous monitoring for treatment failure [25,28]. The perceptions of healthcare providers who act as the gatekeepers between traditional practices and modern evidence-based medicine are decisive in determining the feasibility and safety of such integrated strategies [16,17]. Any policy regarding the use of *Artemisia annua* must therefore be informed by the socio-professional realities of frontline health workers [17,29].

In this context, understanding the perspectives of healthcare workers regarding the integration of *Artemisia annua* tea is paramount for developing effective and acceptable public health interventions. This study endeavors to bridge this gap by exploring the knowledge, attitudes, and practices of healthcare providers in Kalima District concerning the potential role of Artemisia annua tea in uncomplicated malaria treatment.

## 3. Materials and Methods

### 3.1. Study Design and Setting

This study was conducted in the Kalima Health Zone, Maniema Province. Kalima is situated in East Congo, a region characterized by extreme logistical isolation. Essential medicines often travel over **2000 km by road from Nairobi**, the location of the nearest WHO-approved suppliers, or must be procured through complex routes from Kinshasa [30]. Despite the DRC receiving substantial international support estimated at **USD 350 million per year** or roughly 10–12% of the Global Fund’s malaria budget, supply chain disruptions remain chronic [31]. These disruptions are exacerbated by ongoing **political tensions and instability in the neighboring Kivu region**, which directly impact the reliability of the ‘last-mile’ delivery for Artemisinin-based Combination Therapies [30,31].

### 3.2. Study Population and Sampling

The study population included healthcare providers (physicians, nurses, and community health workers) practicing in the health facilities of the Kalima zone, encompassing **Kalima, Bobela, Kakutya 1, Kinkungwa, and Lubile**. Eligible participants were required to have at least six months of professional experience in the area. Providers on long-term leave or those who declined consent were excluded.

Sample Size Determination:

The sample size was determined using Cochran’s formula for infinite populations, a standard approach for cross-sectional surveys to ensure representative results [32]:

$$n=\frac{Z^{2}\cdot P\cdot (1-P)}{d^{2}}$$

where Z=1.96 (95% confidence level), P=0.50 (estimated prevalence to maximize variance), and d=0.05 (margin of error). Although the theoretical calculation required 384 participants, the final sample was adjusted based on the actual pool of available providers in the targeted health areas, resulting in a cohort of 337 participants. This size provides sufficient statistical power to analyze the independent determinants of clinical acceptability [32].

### 3.3. Data Collection Procedures

Data were collected using a structured questionnaire specifically designed for this study. To ensure data integrity and minimize entry errors, the survey was administered through the **KoboCollect** digital platform on mobile devices [33]. Trained independent enumerators conducted interviews in person to ensure consistency across the Kalima health zone. **The survey instrument is available as Supporting Information (Supplement S1)**.

### 3.4. Study Variables

The selection of variables addressed both the socio-economic and clinical dimensions of malaria management.

- **Dependent Variables:** The overall acceptability of *Artemisia annua* tea and the perception of its clinical efficacy.
- **Independent Variables:** Socio-demographic factors (age, sex, education, profession) and clinical knowledge regarding both malaria and the plant, alongside financial and geographical accessibility metrics.

### 3.5. Data Processing and Statistical Analysis

Data were cleaned and analyzed using statistical software. We employed descriptive statistics, including frequencies and percentages, to characterize the study population. For inferential analysis, **Pearson’s Chi-square tests** assessed bivariate associations between provider characteristics and clinical acceptability. Variables found to be significant in the bivariate phase were subsequently entered into a **multivariate logistic regression model**. We selected this method to calculate **Adjusted Odds Ratios (aORs)** while controlling for potential confounders, allowing for the identification of independent predictors of acceptability [16,29]. The threshold for statistical significance was set at p<0.05 , and results are presented with **95% Confidence Intervals (95% CIs)**.

### 3.6. Mitigation of Potential Biases

In accordance with the **STROBE** statement, we implemented several measures to address potential sources of bias [34].

- **Social Desirability Bias**: Given the controversial nature of *Artemisia annua* within official PNLP guidelines, participants might have provided answers they perceived as “professionally correct.” To mitigate this, we ensured strict anonymity, conducted interviews in private settings, and used independent enumerators not affiliated with the health zone’s administrative hierarchy [35,36].
- **Selection Bias**: To ensure the representativeness of the healthcare workforce in Kalima, we employed a systematic sampling strategy across both urban and rural health areas, reaching the required statistical power through a final cohort of 337 providers [32].
- **Information and Recording Bias**: The use of the **KoboCollect** digital platform allowed for real-time data validation, with pre-configured skip patterns and mandatory fields that minimized entry errors and prevented incomplete submissions [37].
- **Recall Bias**: To ensure data accuracy regarding drug stockouts and clinical experiences, the survey focused on events within the last six months and current perceptions of efficacy.

### 3.7. Ethical Considerations

The research protocol was conducted in strict accordance with the ethical principles for medical research involving human subjects as outlined in the Declaration of Helsinki. Formal ethical approval was granted by the **Research Ethics Committee of the Institut Supérieur de Techniques Médicales de Kindu** (Ref: **035/ISTM-KD/C.E.R. I/PRESI/IRBE/2025**, dated 21 August 2025).

Prior to data collection, written informed consent was obtained from all 337 participating healthcare professionals. Each participant received a comprehensive oral and written explanation of the study’s objectives, the methods of data collection via the **KoboCollect** platform, and the intended use of the results. Participants were explicitly informed that their involvement was strictly voluntary and that they maintained the right to withdraw from the study at any time without any professional or personal disadvantage.

Crucially, no financial compensation or incentives were provided to the participants for their involvement, ensuring that their contributions were based solely on voluntary cooperation.

To ensure the highest level of confidentiality and anonymity, no personally identifiable information (such as names or contact details) was recorded in the final analytical dataset. All digital data were encrypted during transmission and stored on a secure, password-protected server accessible only to the principal investigator [33,36]. Administrative authorization was also obtained from the **Maniema Provincial Health Division** and the **Kalima Health Zone Central Office** before the commencement of field activities.

## 4. Results

This study analyzed the responses of 337 healthcare professionals in the Kalima health zone to evaluate the clinical acceptability of integrating *Artemisia annua* tea into the local healthcare system.

### 4.1. Socio-Demographic and Professional Characteristics

The socio-demographic profile of the participants reveals a predominantly male workforce (78.0%), with a majority of providers being married (82.2%). A significant proportion of the respondents (58.2%) practice in rural health areas (Table 1). From a professional standpoint, nurses represent the largest occupational group (31.2%), followed by community health workers (16.0%) and physicians (9.8%).

### 4.2. Knowledge and Perceptions of Artemisia annua

Regarding the preparation of the infusion, the local protocol typically involves an infusion involving **5 g of dried leaves infused in 1 L of water**, with adults consuming the full litre daily for seven days (a total course of **35 g of leaves**) [28]. The clinical knowledge and perceived efficacy of Artemisia annua tea among the participants are summarized in Table 2.

### 4.3. Barriers, Drivers, and Training Requirements

The in-depth analysis of barriers to clinical recommendation reveals an unexpected level of scientific maturity among healthcare providers in Kalima. Although overall acceptability is high, 22.6% ( n=76 ) of providers explicitly identified the **lack of quality control or standardization**, and 18.4% ( n=62 ) cited the **perceived risk of ineffectiveness** as major obstacles to their professional endorsement.

This critical reflection underscores an acute awareness of the challenges surrounding **galenic standardization**. Without a reproducible dosage of artemisinin and its synergistic flavonoids, the use of the whole plant risks inducing sub-therapeutic selection pressures. By expressing these concerns, local providers align with international warnings regarding the potential acceleration of parasite resistance in Sub-Saharan Africa if phytotherapy is not strictly regulated by validated clinical protocols.

Furthermore, beyond theoretical support, a significant proportion of participants (58.8%, n=198 ) reported having already recommended *Artemisia annua* tea to their patients, primarily as a complementary option to conventional therapies (60.5%). This pragmatic use in the field, coupled with a massive demand for specific training (91.4%, n=308 ), suggests that the integration of *Artemisia* into the 2026 National Strategic Plan should not be a mere distribution of seeds, but rather a transition toward pharmaceutical-grade production and evidence-based clinical training [17,38].

The results presented in Table 3 highlights a critical tension between the high clinical acceptability of *Artemisia annua* and the professional commitment to evidence-based medicine among healthcare providers in Kalima. Nearly half of the participants (49.0%) identify the **lack of sufficient clinical evidence** as the primary barrier to recommending the plant, while 44.8% cite the **absence of official recommendations** from the WHO or the National Malaria Control Program. This reliance on institutional validation reflects a professional caution similar to that observed among orthodox practitioners in Ghana, who prioritize regulatory certification and clinical protocols before integrating traditional remedies into formal care.

These barriers align with the broader scientific debate surrounding the variability of artemisinin extraction in aqueous infusions, which ranges from 25% to 90%, potentially exposing parasites to sub-therapeutic concentrations. Such variability remains a significant driver of skepticism, as unstandardized dosing is hypothesized to facilitate the selection of resistant **PfKelch13 markers** already emerging in the Great Lakes region [39].

Crucially, the demand for specific training indicates that healthcare providers do not advocate for informal usage, but rather for a professionalized, evidence-based framework. The preference for training supported by clinical study results and clear protocols underscores the need for “scaffolded” integration, where traditional knowledge is legitimized through pharmaceutical-grade characterization, such as the **ConPhyMP guidelines** [40]. Consequently, addressing these barriers will require a transition from anecdotal practice to a scientifically validated phytopharmaceutical model that ensures both clinical efficacy and health system resilience [41].

### 4.4. Determinants of Clinical Acceptability

The multivariate logistic regression analysis identified several key factors independently associated with the clinical acceptability of integrating *Artemisia annua* tea for the treatment of uncomplicated malaria. **Rural residency** emerged as the strongest independent predictor of acceptability (**aOR = 6.847; 95% CI: 1.944–24.115; *p* = 0.003**). This indicates that healthcare providers practicing in remote areas are nearly seven times more likely to support the integration of the tea than their urban counterparts. This finding reflects a pragmatic response to the structural failures of the conventional health system in rural zones, where geographical isolation and high costs of standard therapies make alternative treatments a clinical priority for health system resilience.

Furthermore, the **perception of the tea’s efficacy** (**aOR = 0.024; 95% CI: 0.007–0.089; *p* < 0.001**) and the **provider’s previous willingness to prescribe** herbal preparations (**aOR = 0.066; 95% CI: 0.015–0.289; *p* < 0.001**) were identified as highly significant determinants. These results confirm that clinical acceptability is primarily driven by the providers’ professional conviction regarding the therapeutic value of the plant and their clinical experience.

The **importance of political involvement** was also found to be a significant factor in shaping provider attitudes toward integration (**aOR = 0.390; 95% CI: 0.110–1.360; *p* = 0.021**), suggesting that institutional support is perceived as a necessary condition for formalizing such treatments. In contrast, demographic variables such as the **sex** of the provider showed no statistically significant association with clinical acceptability (**aOR = 0.550; 95% CI: 0.176–1.721; *p* = 0.304**), indicating that these professional perceptions transcend gender lines within the Kalima health zone.

The multivariate logistic regression analysis (Table 4) confirms that clinical acceptability is primarily driven by geographical context and the provider’s conviction regarding the plant’s therapeutic value. The strong influence of rural residency underscores that logistical challenges in conventional drug supply likely drive the acceptance of plant-based alternatives as a pragmatic solution for health system resilience.

## 5. Discussion

The analysis of data collected in the Kalima health zone highlights a profound divergence between local clinical realities and the international regulatory frameworks governing malaria control. The Democratic Republic of the Congo is a “High Burden High Impact” country, representing **12.4% of the global malaria burden** [2]. With a population of **96 million**, all of whom are at risk, the scale of the tragedy is evidenced by an estimated **29.88 million malaria cases** annually [42]. While WHO modeling suggests a case fatality rate of approximately **1:333**, the gap between an estimated **73,000 deaths** and only **21,300 reported deaths** suggests that systemic barriers to care place the DRC in a significantly worse condition than official figures indicate [43,44].

This study is among the first to quantify, on such a significant scale ( N=337 ), the perceptions of healthcare providers in the Democratic Republic of the Congo, offering unique insights into the resilience mechanisms of local health systems facing logistical and epidemiological challenges

### 5.1. Divergence Between Local Adherence and WHO Guidelines

It is essential to clarify that the high level of clinical acceptability (81.0%) observed in this study reflects the professional perceptions and pragmatic attitudes of healthcare workers in a resource-limited setting, rather than a clinical validation of the tea’s efficacy or safety. These findings should be interpreted as a public health indicator of health system resilience, not as a medical endorsement of non-pharmaceutical preparations [17,45].

The marked contrast between the World Health Organization’s restrictive position and the widespread clinical adoption of *Artemisia annua* infusions in the Kalima health zone highlights a profound “policy-practice gap”. While international guidelines prioritize the prevention of artemisinin resistance through the use of standardized Artemisinin-based Combination Therapies, local healthcare providers often resort to traditional preparations as a pragmatic response to frequent stockouts and high costs of conventional therapies, particularly in remote areas. This situation transforms geographical and financial accessibility into clinical priorities, underscoring the role of traditional medicine as a first line of defense when conventional drug supplies are interrupted. A major finding of this study is the striking contrast between the high local acceptability of integration (81.0%) and the restrictive position of the World Health Organization. Since 2012, the WHO has formally opposed the use of *Artemisia annua* plant material for malaria treatment [46]. This stance is driven by the risk that herbal infusions do not guarantee a stable or sufficient concentration of artemisinin to ensure total parasite clearance, which can lead to therapeutic failure or recrudescence [46]. However, our results indicate that 82.8% of providers in Kalima judge the tea to be effective (Table 2). This perception, while purely observational, suggests that the use of the plant has established itself as a default “therapeutic pressure” solution in areas where access to ACTs is limited [10,38].

### 5.2. Resistance Risks and the Imperative of Galenic Standardization

The “scientific mistrust” expressed by 39.8% of participants (Table 3) aligns with major global concerns regarding the emergence of artemisinin resistance in Africa [3,8]. The documented emergence of mutations in the Kelch 13 gene—such as **R561H** in East Africa, **P441L** in Southern Africa, and the recent detection of **C469Y** and **A675V** in Western Kenya—is directly correlated with delayed parasite clearance after treatment [4,6,39,47].

While synergistic effects are hypothesized (pACT), the inherent variability in artemisinin extraction—ranging from 25% to 90% in aqueous infusions—remains the critical scientific bottleneck [48]. Without standardized phytochemical characterization, as recommended by the ConPhyMP guidelines, any informal use risks exposing parasites to sub-therapeutic concentrations, potentially fostering the selection of resistant PfK13 markers, which are already emerging in the region [7,39,40].

This dosage variability—ranging from 25% to 90% in aqueous infusions [48]—is the primary argument for opponents of phytotherapy, as unstandardized exposure could accelerate the loss of efficacy of essential antimalarial molecules, fostering subtherapeutic concentrations that select for resistant *Plasmodium falciparum* strains such as those harboring *kelch13* mutations (e.g., R561H, C469Y, A675V) already emerging across Africa [3,8,39] situation with potentially catastrophic consequences for the Democratic Republic of the Congo, which bears 12.4% of the global malaria burden [2,42]. Consequently, transitioning from “informal use” to “clinical integration” must be contingent upon pharmaceutical-grade production, rigorous phytochemical standardization per ConPhyMP guidelines [40], and evidence-based clinical training [49]. The widespread use of *Artemisia annua* tea in Africa, particularly without standardized preparation, raises concerns about subtherapeutic exposure and the potential selection of resistant parasite strains, which could lead to artemisinin resistance. This risk is exacerbated by the diverse strains of *Artemisia annua* or *Artemisia afra* used in informal trials, making it difficult to standardize the artemisinin dosage within each treatment [28].

### 5.3. Socio-Economic Determinants: Rurality as a Driver of Acceptability

The originality of this research also lies in identifying the predictive factors of acceptability through multivariate analysis. The fact that rural healthcare providers are 6.8 times more likely to accept the integration of the tea emphasizes that acceptability is, above all, a pragmatic response to systemic failures. In rural areas, the high cost of ACTs and frequent supply chain disruptions—exacerbated by the Democratic Republic of the Congo’s 12.4% share of the global malaria burden [2,42] transform geographical and financial accessibility into absolute clinical priorities [31,50,51]. This study demonstrates that phytotherapy is perceived not only as a cultural heritage but as a tool for resilience in the face of a precarious conventional health system, particularly amid extreme logistical failures such as the Kalima health zone’s remoteness, approximately 2000 km by road from Nairobi, the nearest hub for WHO-approved suppliers—a route frequently disrupted by ongoing armed conflicts in eastern DRC, which impose roadblocks, restrict access, and cause delivery delays leading to drug stockouts [9]. The standard local protocol involves a decoction of 5 g of dried leaves infused in 1 L of water, consumed daily for seven days. The economic driver is clear: while a complete treatment of 35 g of dried leaves costs approximately USD 0.60 through organized networks like La Maison de l’Artemisia, prices on the private market can be ten times higher, making the tea a pragmatic alternative during ACT stockouts [50,51]. To mitigate resistance risks amid emerging Pfkelch13 mutations across Africa [3,8] it imperative to implement genomic surveillance using Whole Genome Sequencing in cases of treatment failures [3,52].

To address these concerns, this study is being followed by a comprehensive genomic surveillance investigation in the same study area. This ongoing research employs whole genome sequencing to compare the prevalence of *Plasmodium falciparum* kelch13 resistance markers among three distinct patient groups: individuals using *Artemisia annua* tea for curative treatment of uncomplicated malaria, individuals using the tea for preventive purposes, and patients treated with standard Artemisinin-based Combination Therapies. This molecular surveillance will provide essential evidence to determine whether informal tea consumption contributes to differential selection of resistant parasite strains in the Maniema province, thereby defining safety parameters for future phytopharmaceutical integration [3,52,53].

### 5.4. Training Needs and Regulatory Frameworks

The near-unanimous desire for specific training (91.4%, Table 2) reveals that providers in Kalima aspire to move from informal practice toward a structured and secure framework. Their expectations focus on dosage and biological modes of action (Table 3), areas where academic research must still provide standardized protocols to ensure patient safety [49,54]. Successful integration, as attempted in certain frameworks in Ghana, shows that without rigorous protocols and strong political commitment, the real impact on disease control remains limited [55]. Our results confirm that the absence of official recommendations (44.8%) is the major barrier to safe prescription (Table 3) [56].

### 5.5. Originality, Strengths, and Limitations of the Study

This study is distinguished by its methodological robustness, relying on a representative sample of 337 healthcare professionals—including physicians, nurses, and pharmacy managers—within the Kalima district, thereby providing high statistical power for the analysis of clinical acceptability determinants [32]. The originality of this research lies in its “field-based” approach within a hyper-endemic area of Maniema, capturing the tensions between institutional directives and the practical reality of ACT stockouts [1,31]. The integration of the KoboCollect digital platform ensured high data integrity and minimized recording errors, representing a major technical strength for research conducted in remote rural settings in the DRC [33,36,57]. To ensure scientific neutrality and mitigate potential investigator bias, independent enumerators conducted data collection, and the analysis focused strictly on quantifiable professional determinants [33,36]. This study deliberately avoids clinical advocacy, focusing instead on documenting the ‘reality gap’ between international guidelines and field practices in Maniema.

However, in accordance with the STROBE statement recommendations, several limitations must be acknowledged [58]:

- **Social Desirability Bias:** Given that *Artemisia annua* infusion is not yet subject to official recommendation by the PNLP, some participants may have under-reported their actual adherence to align with formal protocols [59]. We mitigated this bias by ensuring strict anonymity, conducting interviews in private settings, and employing independent enumerators not affiliated with the health zone’s administrative hierarchy. Furthermore, to ensure scientific neutrality and mitigate potential investigator bias, the analysis focused strictly on quantifiable professional determinants, deliberately avoiding clinical advocacy and focusing instead on documenting the ‘reality gap’ between international guidelines and field practices in Maniema.
- **Selection Bias:** To ensure the representativeness of the healthcare workforce in Kalima, we employed a systematic sampling strategy across both urban and rural health areas, reaching the required statistical power through a final cohort of 337 providers [60].
- **Information and Recording Bias:** The use of the KoboCollect digital platform allowed for real-time data validation, with pre-configured skip patterns and mandatory fields that minimized entry errors and prevented incomplete submissions [61].
- **Recall Bias:** Certain data regarding past drug stockouts or prior clinical experiences relied on the participants’ memory. To minimize this risk, the recall period was restricted to the six months preceding the survey [62].
- **Generalizability:** While our findings provide crucial insights into the Maniema province, the high acceptability rate (81.0%) reflects a specific regional reality characterized by extreme health system fragility. These conclusions may not be directly generalizable to urban areas with more stable ACT supply chains.

Despite these limitations, this study provides an essential evidence base for the upcoming 2026 National Strategic Plan, advocating for the structured and secure integration of traditional pharmacopeia into the formal health sector.

### 5.6. Regional Emergence of PfKelch13 Mutations and Clinical Implications

The high acceptability of *Artemisia annua* tea in Kalima must be evaluated within the context of the rapidly shifting molecular landscape of malaria in the Great Lakes region. Recent surveillance (2024–2025) has confirmed a significant upsurge in *Plasmodium falciparum* kelch13 (*Pfk13*) mutations—molecular markers of artemisinin partial resistance—in neighboring Rwanda, Uganda, and Tanzania [7]. In Uganda, the selection of these mutations is occurring at rates comparable to the early stages of resistance emergence in Southeast Asia [63]. While these mutations have only recently been detected at lower prevalence in the Democratic Republic of the Congo [64], the geographical proximity to these “resistance hotspots” creates an urgent need for clinical vigilance [65]. Using non-standardized herbal preparations in a region where delayed parasite clearance is already emerging could inadvertently facilitate the selection of more resilient strains [7,23].

To further investigate the biological implications of these professional perceptions, a complementary molecular study is currently underway in the same health zone. This ongoing research compares the prevalence of PfKelch13 resistance markers among patients using Artemisia annua tea versus those treated with standard ACTs. Genomic analysis is being conducted to determine if informal tea consumption contributes to a differential selection of resistant parasite strains (such as **R561H**) in the Maniema province, providing the first direct evidence of its kind in this hyper-endemic region [6,52,53]. This work will specifically examine whether the widespread, unregulated use of Artemisia annua tea in Kalima correlates with an increased frequency of specific PfKelch13 mutations associated with artemisinin resistance, thereby providing crucial insights for regional public health policy [3].

### 5.7. Comparative Analysis: Lessons from Ghana and Kenya’s Integrated Models

The high level of clinical acceptability (81.0%) observed in the Kalima health district suggests that the Democratic Republic of the Congo is uniquely positioned to follow the path of other Sub-Saharan nations that have pioneered the integration of herbal medicine into formal healthcare. Specifically, the **Ghanaian model** provides a critical benchmark. Ghana formally integrated herbal medicine into its primary healthcare system in 2012, establishing herbal units in over 18 public hospitals staffed by university-trained **Medical Herbalists** [17,66]. This integration was supported by the creation of the **Traditional Medicine Practice Council** and the publication of the **Ghana Herbal Pharmacopoeia** in 2007, which provided the necessary regulatory and standardization framework that providers in Kalima currently require [17].

However, the Ghanaian experience also reveals significant challenges that the DRC must anticipate. Recent studies indicate that despite a decade of integration, medical doctors in Ghana remain skeptical about the impact of herbal antimalarials on malaria control due to inconsistent supply chains and a perceived lack of standardization in practice. This mirrors the **scientific skepticism (39.8%)** and the demand for clear clinical protocols (26.4%) expressed by providers in our study. In both contexts, inadequate political commitment and the high cost of regulated herbal medications widen the “policy-practice gap”, which can lead patients back to informal, unstandardized markets.

Similarly, **Kenya** has made significant strides by including traditional medicine in its **National Pharmaceutical Policy** since 2008 and enacting the **Health Act of 2017**, which provides a legal mandate for the registration and standardization of herbal products [67,68]. Like the providers in Kalima who view *Artemisia annua* as a tool for resilience against ACT stockouts, the Kenyan model aims to mainstream traditional medicine to boost universal health access. Yet, Kenya still struggles with slow regulatory enforcement and a fragmented oversight system where certificates of recognition are sometimes issued by non-health ministries, undermining the clinical authority of the practice.

For the DRC, the lesson from these models is clear: **acceptability alone is insufficient for successful integration**. The near-unanimous demand for training (91.4%) in our study indicates that, like their counterparts in Ghana and Kenya, Congolese providers require a **scientifically validated phytopharmaceutical model** [17,67]. The establishment of a national pharmacopoeia and the training of specialized personnel who can bridge the gap between botanical synergy and pharmaceutical rigor must precede integration [40]. Without this institutional “scaffolding,” the DRC risks repeating the pitfalls of informal usage, which could ultimately compromise malaria elimination efforts through sub-therapeutic dosing and the acceleration of **PfKelch13 resistance** [6,14,48].

### 5.8. Recommendations for Public Health Policy in the DRC

The high clinical acceptability (81.0%) of *Artemisia annua* in Maniema is not merely a cultural preference but a pragmatic response to health system fragility. To bridge the gap between local practice and global health security, we propose a four-pillar strategic framework for the Democratic Republic of the Congo:

1. **Establishment of a National Regulatory Framework for Phytopharmaceuticals:** The DRC should accelerate the integration of traditional medicine into the national healthcare system by developing a formal **National Pharmacopoeia** [25,26]. Following the Ghanaian model, the Ministry of Health must establish clear registration pathways for standardized *Artemisia* preparations, ensuring they are recognized as “complementary medicines” rather than informal remedies [17,66]. This framework should define the legal boundaries of their use, specifically restricting them to uncomplicated malaria cases while mandating strict adherence to conventional diagnostic protocols [38,41].
2. **Implementation of Stringent Galenic Standardization:** To mitigate the risk of sub-therapeutic dosing which can accelerate the selection of **PfKelch13 resistance markers** all *Artemisia*-based products must undergo rigorous quality control [6,48]. We recommend adopting the **ConPhyMP guidelines** for the phytochemical characterization of extracts to ensure consistent artemisinin and flavonoid content [40,41]. Transitioning from “tea infusions” to standardized, encapsulated, or pharmaceutical-grade preparations is a *sine qua non* condition for institutionalizing their use in public facilities [40,48].
3. **Integration of Genomic Surveillance into Clinical Trials:** Given the detection of **R561H** mutations in the DRC, future clinical validation of *Artemisia* must be paired with molecular monitoring [52,53]. We recommend prioritizing large-scale, multi-center clinical trials that evaluate not only parasite clearance times but also the long-term impact on resistance selection [7,69]. The ongoing genomic research initiated in this health zone should be scaled up to monitor the biological safety of both ACTs and botanical alternatives [14,39,52].
4. **Specialized Training and Capacity Building:** The near-unanimous demand for training (91.4%) highlights a significant knowledge gap. The National Malaria Control Program should develop integrated training modules for healthcare providers, focusing on the biological mode of action, standardized dosing, and the risks of resistance [17]. By professionalizing the practice, the DRC can transform healthcare providers from passive observers of informal use into active gatekeepers of a scientifically validated, pluralistic health system [27,38,70].

Ultimately, these recommendations aim to harness local botanical resources to strengthen health system resilience without compromising artemisinin’s therapeutic life [71]. By moving from a policy of exclusion to one of **regulated integration**, the DRC can address the chronic shortages of conventional treatments while actively contributing to global malaria elimination goals. This strategic approach, therefore, transforms the high acceptability of Artemisia annua in Kalima from a localized health practice into a significant public health opportunity, allowing the DRC to proactively manage resistance while enhancing malaria response capabilities.

## 6. Conclusions

This study investigated the professional determinants of the “policy–practice gap” regarding *Artemisia annua* tea in the Kalima health district, Maniema, DRC. Our findings demonstrate a high prevalence of clinical acceptability (**81.0%**) among healthcare providers, which is independently and strongly predicted by **rural residency (aOR = 6.847; 95% CI: 1.944–24.115)**. This indicates that the integration of herbal infusions is not a rejection of modern medicine but a pragmatic response to systemic failures, including frequent ACT stockouts and high treatment costs [38].

However, this adherence is tempered by a significant level of scientific caution among frontline providers. Despite their use of the plant, **39.8%** expressed mistrust regarding the current informal use of infusions, citing concerns over unstandardized dosing. This professional apprehension is scientifically grounded; the documented variability in artemisinin extraction—ranging from **25% to 90%** in aqueous infusions—poses a direct risk of sub-therapeutic exposure [48]. In a regional context where **PfKelch13 resistance markers** (such as **R561H, C469Y, and A675V**) are already emerging, ensuring repeatable therapeutic concentrations is an absolute prerequisite for any formal integration [3,6,39]. To assist further investigations and policy development, our results underscore the need for a transition toward a **phytopharmaceutical model**. Drawing on the “Ghanaian model” of registering standardized herbal products as “complementary medicines,” the DRC could formalize these practices through a national regulatory framework and the establishment of a formal pharmacopoeia [17,25,40]. Future research must prioritize evaluating the stability of active ingredients in pharmaceutical-grade preparations and utilizing the ongoing **National Genomic Surveillance Project** to compare the molecular signatures of *Plasmodium falciparum* between *Artemisia* tea users and patients treated with conventional ACTs [52,53,72].

Ensuring that any integration of Artemisia-based infusions is guided by rigorous pharmacological standardization and robust clinical evidence will be essential to safeguard artemisinin efficacy for future generations in the DRC.

## Figures and Tables

**Table 1:** Socio-demographic and Professional Characteristics of Healthcare Providers ( N=337 ).

Characteristics	Frequency (*n*)	Percentage (%)
**Sex**		
Male	263	78.0
Female	74	22.0
**Residence Setting**		
Rural	196	58.2
Urban	141	41.8
**Marital Status**		
Married	277	82.2
Single	29	8.6
Other (Widowed/Divorced)	31	9.2
**Professional Category**		
Nurse	105	31.2
Health Worker	54	16.0
Physician	33	9.8
Midwife	31	9.2
Others	114	33.8

**Commentary:** The data indicate that the healthcare workforce in the Kalima zone is characterized by a strong masculine presence and is largely distributed across rural health areas. Nurses and community health workers form the backbone of the primary care response, highlighting their critical role as potential agents for any integrated malaria control strategy.

**Table 2:** Clinical Knowledge and Perceived Efficacy of *Artemisia annua* Tea.

Variables	Frequency (*n*)	Percentage (%)
**Perceived Efficacy of the Tea**		
Effective	279	82.8
Not effective/Do not know	58	17.2
**Self-Reported Competence**		
Sufficient	154	45.7
Partially sufficient	78	23.1
Insufficient	105	31.2
**Provider Perception of Patient Knowledge**		
Very Good/Good	166	49.3
Average	108	32.0
Low/Very Low	63	18.7

**Commentary:** These findings reveal a notable discrepancy between the providers’ belief in the treatment’s efficacy and their perceived clinical competence. This suggests that while there is strong professional interest in the plant, the lack of formal training may pose a risk to standardized clinical application.

**Table 3:** Barriers to Clinical Recommendation and Training Requirements ( N=337 ).

Variables	Frequency (*n*)	Percentage (%)
**Major Barriers to Recommendation**		
Lack of sufficient clinical evidence	165	49.0
Absence of official recommendations (NMCP/WHO)	151	44.8
**Absence of quality control/standardization**	**76**	**22.6**
**Perceived risk of ineffectiveness**	**62**	**18.4**
Administrative and regulatory constraints	77	22.8
Risk of parasite resistance development	48	14.2
**Origin of Perceived Resistance**		
Scientific mistrust/Skepticism	134	39.8
Preference for conventional therapy	99	29.4
Fear of side effects or complications	56	16.6
**Training and Integration Needs**		
**Desire for specific training on Artemisia**	**308**	**91.4**
Preferred support: Results of clinical studies	124	36.8
Preferred support: Practical training sessions	112	33.2
Preferred support: Clear clinical protocols	89	26.4

**Commentary on Table 3: Barriers to Clinical Recommendation and Training Requirements.**

**Table 4:** Multivariable logistic regression Determinants of clinical acceptability (N = 337). Dependent variable: willingness to prescribe or recommend the herbal tea if validated (Yes = 1; No+Don’t know = 0).

Predictor	Adjusted OR	95% CI	Sig. (*p*)
Constant	0.333	[0.074–1.439]	0.1445
Sex: Female (ref. Male)	0.545	[0.253–1.184]	0.1210
Area: Rural (ref. Urban)	0.608	[0.245–1.409]	0.2615
Perceived efficacy: Yes (ref. No)	26.490	[11.331–68.315]	<0.001 ***
Perceived political involvement	1.557	[1.022–2.394]	0.0406 *
Barrier: Administrative constraints	0.293	[0.113–0.739]	0.0099 **
Barrier: Concerns about resistance	0.803	[0.251–2.748]	0.7180
Barrier: Safety concerns	0.243	[0.082–0.730]	0.0108 *
Barrier: Rejection by patients	0.205	[0.060–0.722]	0.0117 *

* *p* < 0.05; ** *p* < 0.01; *** *p* < 0.001. ref. = reference category.

## Data Availability

All relevant data are within the manuscript and its Supplementary Materials. The raw, anonymized dataset used for the statistical analysis (Cochran formula-based sample of N = 337) is provided as Data S1. The survey instrument used for data collection in the Kalima Health Zone is included as Supplementary S1. Any additional inquiries can be directed to the corresponding author or the ethics committee of the Research Ethics Committee of the Institut Supérieur de Techniques Médicales de Kindu.

## Supplementary Materials

The following supporting information can be downloaded at: https://www.mdpi.com/article/10.3390/tropicalmed11040105/s1.

## Abbreviations

List of abbreviations and acronyms.

**Abbreviation**	**Meaning (English/French)**
**ACT/TCA**	Artemisinin-based Combination Therapy/Combinaison Thérapeutique à base d’Artémisinine
**AOR/ORA**	Adjusted Odds Ratio/Odds Ratio Ajusté
**BCZ**	Health Zone Central Office/Bureau Central de Zone de Santé
**CS**	Health Center/Centre de Santé
**95% CI/IC 95%**	95% Confidence Interval/Intervalle de Confiance à 95%
**DLA**	Dried leaf *Artemisia annua*/Feuilles séchées d’*Artemisia annua*
**DPS**	Provincial Health Division/Division Provinciale de la Santé
**DRC/RDC**	Democratic Republic of the Congo/République Démocratique du Congo
**HGR**	General Reference Hospital/Hôpital Général de Référence
**MCZ**	Health Zone Chief Medical Officer/Médecin Chef de Zone
**NMCP/PNLP**	National Malaria Control Program/Programme National de Lutte contre le Paludisme
**OR**	Odds Ratio/Rapport des Cotes
**PfK13**	*Plasmodium falciparum* kelch 13/Marqueur de résistance
**RDT/TDR**	Rapid Diagnostic Test/Test de Diagnostic Rapide
**WHO/OMS**	World Health Organization/Organisation Mondiale de la Santé
***p*** **-value**	Statistical significance value/Valeur de significativité statistique

## References

[1] Venkatesan P. (2025). WHO world malaria report 2024. Lancet Microbe.

[2] Deutsch-Feldman M., Parr J.B., Keeler C., German M.S., Plucinski M.M., Filler S.J., Keating J., Kadjunga A., Mwandagalirwa M.K., Havumaki J.S. (2020). The burden of Malaria in the democratic Republic of the Congo. J. Infect. Dis..

[3] Rosenthal P.J., Asua V., Bailey J.A., Conrad M.D., Ishengoma D.S., Kamya M.R., Rasmussen C., Tadesse F.G., Uwimana A., A Fidock D. (2024). The emergence of artemisinin partial resistance in Africa: How do we respond?. Lancet Infect. Dis..

[4] Rosenthal P.J., Asua V., Conrad M.D. (2024). Emergence, transmission dynamics and mechanisms of artemisinin partial resistance in malaria parasites in Africa. Nat. Rev. Microbiol..

[5] Schreidah C., Giesbrecht D., Gashema P., Young N.W., Munyaneza T., Muvunyi C.M., Thwai K., Mazarati J.-B., Bailey J.A., Juliano J.J. (2024). Expansion of artemisinin partial resistance mutations and lack of histidine rich protein-2 and -3 deletions in *Plasmodium falciparum* infections from Rukara, Rwanda. Malar. J..

[6] Uwimana A., Legrand E., Stokes B.H., Ndikumana J.-L.M., Warsame M., Umulisa N., Ngamije D., Munyaneza T., Mazarati J.-B., Munguti K. (2020). Emergence and clonal expansion of in vitro artemisinin-resistant Plasmodium falciparum kelch13 R561H mutant parasites in Rwanda. Nat. Med..

[7] Assefa A., Fola A.A., Tasew G. (2024). Emergence of Plasmodium falciparum strains with artemisinin partial resistance in East Africa and the Horn of Africa: Is there a need to panic?. Malar. J..

[8] Ishengoma D.S., Gosling R., Martinez-Vega R., Beshir K.B., Bailey J.A., Chimumbwa J., Sutherland C., Conrad M.D., Tadesse F.G., Juliano J.J. (2024). Urgent action is needed to confront artemisinin partial resistance in African malaria parasites. Nat. Med..

[9] Altare C., Castelgrande V., Tosha M., Malembaka E.B., Spiegel P. (2021). From Insecurity to Health Service Delivery: Pathways and System Response Strategies in the Eastern Democratic Republic of the Congo. Glob. Health Sci. Pract..

[10] Ridder Sde Kooy Fvan der Verpoorte R. (2008). Artemisia annua as a self-reliant treatment for malaria in developing countries. J. Ethnopharmacol..

[11] Ekiert H., Świątkowska J., Klin P., Rzepiela A., Szopa A. (2021). Artemisia annua—Importance in Traditional Medicine and Current State of Knowledge on the Chemistry, Biological Activity and Possible Applications. Planta Medica.

[12] Rasoanaivo P., Wright C.W., Willcox M., Gilbert B. (2011). Whole plant extracts versus single compounds for the treatment of malaria: Synergy and positive interactions. Malar. J..

[13] Xu F., Shan X., Li J., Yuan J., Zou D., Wang M. (2025). The plant matrix of Artemisia annua L. for the treatment of malaria: Pharmacodynamic and pharmacokinetic studies. PLoS ONE.

[14] Ataba E., Dorkenoo A.M., Nguepou C.T., Bakai T., Tchadjobo T., Kadzahlo K.D., Yakpa K., Atcha-Oubou T. (2021). Potential Emergence of Plasmodium Resistance to Artemisinin Induced by the Use of Artemisia annua for Malaria and COVID-19 Prevention in Sub-African Region. Acta Parasitol..

[15] Elfawal M.A., Towler M.J., Reich N.G., Weathers P.J., Rich S.M. (2015). Dried whole-plant Artemisia annua slows evolution of malaria drug resistance and overcomes resistance to artemisinin. Proc. Natl. Acad. Sci. USA.

[16] Hien D., Kaboré J.M.T., Siribié M., Soulama I., Barry N., Baguiya A., Tiono A.B., Tchouatieu A.-M., Sirima S.B. (2022). Stakeholder perceptions on the deployment of multiple first-line therapies for uncomplicated malaria: A qualitative study in the health district of Kaya, Burkina Faso. Malar. J..

[17] Ampomah I.G., Ampomah G.A., Emeto T.I. (2024). Integrating modern and herbal medicines in controlling malaria: Experiences of orthodox healthcare providers in Ghana. Arch. Public Health.

[18] O’Boyle S., Bruxvoort K., Ansah E., Burchett H.E.D., Chandler C.I.R., Clarke S.E., Goodman C., Mbacham W., Mbonye A.K., Onwujekwe O.E. (2020). Patients with positive malaria tests not given artemisinin-based combination therapies: A research synthesis describing under-prescription of antimalarial medicines in Africa. BMC Med..

[19] Abenwie S.N., Shinyuy L.M., Noukimi S.F., Njong S., Bambara S., Kalimba E.M., Kamga J., Ghogomu S.M., Frederich M., Talom J.L.L. (2023). Knowledge about Asymptomatic Malaria and Acceptability of Using Artemisia afra Tea among Health Care Workers (HCWs) in Yaoundé, Cameroon: A Cross-Sectional Survey. Int. J. Environ. Res. Public Health.

[20] Mokuolu O.A., Bolarinwa O.A., Opadiran O.R., Ameen H.A., Dhorda M., Cheah P.Y., Amaratunga C., de Haan F., Tindana P., Dondorp A.M. (2023). A framework for stakeholder engagement in the adoption of new anti-malarial treatments in Africa: A case study of Nigeria. Malar. J..

[21] Adewole A., Ajumobi O., Waziri N., Umar A.A., Bala U., Gidado S., Ugbenyo G., Simple E., Igbaver I., Attahiru A. (2023). Malaria Frontline Project: Strategic approaches to improve malaria control program leveraging experiences from Kano and Zamfara States, Nigeria, 2016–2019. BMC Health Serv. Res..

[22] Yeboah S.N., Ansong M., Clotworthy D., Domie P.E., Chirawurah J.D., Awiaga C.K., Mensah C., Sakyi M.-L.E., Adika E., Morang’a C.M. (2025). The efficacy of selected Ghanaian herbal antimalarials against laboratory strains and clinical isolates of Plasmodium falciparum. Sci. Rep..

[23] Conrad M.D., Asua V., Garg S., Giesbrecht D., Niaré K., Smith S., Namuganga J.F., Katairo T., Legac J., Crudale R.M. (2023). Evolution of Partial Resistance to Artemisinins in Malaria Parasites in Uganda. N. Engl. J. Med..

[24] Ferreira J., Luthria D.L., Sasaki T., Heyerick A. (2010). Flavonoids from Artemisia annua L. as Antioxidants and Their Potential Synergism with Artemisinin against Malaria and Cancer. Molecules.

[25] Thamizhoviya G. (2025). Global Integration of Traditional and Modern Medicine: Policy Developments, Regulatory Frameworks, and Clinical Integration Model. Future Integr. Med..

[26] Ikhoyameh M., Okete W.E., Ogboye R.M., Owoyemi O.K., Gbadebo O.S. (2024). Integrating traditional medicine into the African healthcare system post-Traditional Medicine Global Summit: Challenges and recommendations. Pan Afr. Med. J..

[27] Leonti M., Casu L. (2013). Traditional medicines and globalization: Current and future perspectives in ethnopharmacology. Front. Pharmacol..

[28] Baxerres C., Marquis C. (2018). Regulations, Markets, Health: Questioning Current Stakes of Pharmaceuticals in Africa. Proceedings. HAL (Le Centre pour la Communication Scientifique Directe). https://hal.science/hal-01988227.

[29] Bulafu D., Tamale B.N., Ninsiima L.R., Baguma J.N., Namakula L.N., Niyongabo F., Lubega G.B., Aruhomukama D., Ndejjo R., Musoke D. (2023). Adherence to malaria treatment guidelines among health care workers in private health facilities in Kampala’s informal settlements, Uganda. PLoS Glob. Public Health.

[30] Karim A., Burri C., Kila J.S.N., Bambwelo N., Bakukulu J.T., de Savigny D. (2024). Systems Thinking for Supply Chains: Identifying Bottlenecks Using Process Mapping of a Child Health Intervention in the Democratic Republic of the Congo (DRC). Systems.

[31] Lechthaler F., Matthys B., Lechthaler-Felber G., Likwela J.L., Mavoko H.M., Rika J.M., Mutombo M.M., Ruckstuhl L., Barczyk J., Shargie E. (2019). Trends in reported malaria cases and the effects of malaria control in the Democratic Republic of the Congo. PLoS ONE.

[32] Ryan T.P. (2013). Sample Size Determination and Power.

[33] Lakshminarasimhappa M.C. (2021). Web-Based and Smart Mobile App for Data Collection: Kobo Toolbox/Kobo Collect. https://www.kobotoolbox.org/.

[34] Elm Evon Altman D.G., Egger M., Pocock S.J., Gøtzsche P.C., Vandenbroucke J.P. (2007). The Strengthening the Reporting of Observational Studies in Epidemiology (STROBE) Statement: Guidelines for Reporting Observational Studies. PLoS Med..

[35] Bédia-Tanoh V.A., Angora E.K., Miezan S., Koné-Bravo E.D.M., Konaté-Touré A., Bosson-Vanga H., Kassi F.K., Kiki-Barro P.C.M., Djohan V., Menan H.E.I. (2023). Knowledge and practices of private pharmacy auxiliaries on malaria in Abidjan, Côte d’Ivoire. Malar. J..

[36] Othman O.J., Mashayo E., Jones J., Shah K., Graham C., Yong A.C., Graham R., Omar F., Chan V.F. (2024). Does electronic data collection perform better than paper-based data collection in health research fieldwork? A participatory action research in Zanzibar. BMJ Public Health.

[37] Bello I.M., Umar A., Akpan G.U., Okeibunor J., Shibeshi C., Eshetu M., Magwati C.J., Fasil T., Fussum D., Mihigo R. (2021). Implementation of Mobile Phone Data Collection in the Conduct EPI Comprehensive Review in East and Southern African Countries. J. Immunol. Sci..

[38] Graz B., Kitua A., Malebo H.M. (2011). To what extent can traditional medicine contribute a complementary or alternative solution to malaria control programmes?. Malar. J..

[39] Osoti V., Wamae K., Musau M.M., Magudha J.B., Ndwiga L., Gichuki P.M., Okoyo C., Rosebella K., Mahugu S., Aricha S. (2025). Serial cross-sectional school surveys identifies C469Y, P553L, R561H and A675V kelch 13 mutations associated with artemisinin resistance in Western Kenya. Sci. Rep..

[40] Heinrich M., Jalil B., Abdel-Tawab M., Echeverria J., Kulić Ž., McGaw L.J., Pezzuto J.M., Potterat O., Wang J.-B. (2022). Reporting guidelines for medicinal plant extracts used in pharmacological and toxicological research: ConPhyMP. Br. J. Pharm..

[41] Heinrich M., Jalil B. (2023). From the CONSORT to the ConPhyMP statement and beyond—How to ascertain best practice. Front. Pharmacol..

[42] Alhassan A., Ajala L.S., Ode B., Alanjiro M., Rehman S., Onesime J., Kihanduka E., Tague C., Farhan K., Banga S. (2024). Call for elimination program of Malaria among children under 5 years old living in refugee camps in eastern Democratic Republic of Congo. New Microbes New Infect..

[43] Akilimali A., Mufungizi I., Rasheed S., Tague C., Shams Z., Shah R.J., Wilczyńska W., Siddiqui H.T., Atif M.H., Tufail Z. (2025). Malaria in the Democratic Republic of Congo: How support programs consolidate elimination gains and protect at-risk populations from recrudescence. Ann. Med. Surg..

[44] Danquah J.B., Yamoah J.A.A. (2024). One Health Perspective of Malaria Transmission. Infectious Diseases.

[45] Wilmot D., Kumi A.K., Kwaku O.Y. (2023). Antimalarial Health Seekers’ Preferences and Perceptions: Insights from Ghana. J. Infect. Dis. Epidemiol..

[46] Angupale J.R., Tusiimire J., Ngwuluka N.C. (2023). A review of efficacy and safety of Ugandan anti-malarial plants with application of RITAM score. Malar. J..

[47] Martin A., Sadler J.M., Simkin A., Musonda M., Katowa B., Matoba J., Schue J., Simulundu E., A Bailey J., Moss W.J. (2025). Emergence and Rising Prevalence of Artemisinin Partial Resistance Marker Kelch13 P441L in a Low Malaria Transmission Setting in Southern Zambia. medRxiv.

[48] Suberu J., Gorka A.P., Jacobs L., Roepe P.D., Sullivan N., Barker G.C., Lapkin A.A. (2013). Anti-Plasmodial Polyvalent Interactions in *Artemisia annua* L. Aqueous Extract—Possible Synergistic and Resistance Mechanisms. PLoS ONE.

[49] Dalamagka M.I. (2024). Integrating traditional medicine into a modern health care system. Int. J. Sci. Res. Arch..

[50] Kayiba N.K., Yobi D.M., Devleesschauwer B., Mvumbi D.M., Kabututu P.Z., Likwela J.L., Kalindula L.A., DeMol P., Hayette M.-P., Mvumbi G.L. (2021). Care-seeking behaviour and socio-economic burden associated with uncomplicated malaria in the Democratic Republic of Congo. Malar. J..

[51] Mpanya G., Tshefu A., Likwela J.L. (2017). The malaria testing and treatment market in Kinshasa, Democratic Republic of the Congo, 2013. Malar. J..

[52] Kayiba N.K., Tshibangu-Kabamba E., Rosas-Aguirre A., Kaku N., Nakagama Y., Kaneko A., Makaba D.M., Malekita D.Y., Devleesschauwer B., Likwela J.L. (2023). The landscape of drug resistance in Plasmodium falciparum malaria in the Democratic Republic of Congo: A mapping systematic review. Trop. Med. Health.

[53] Kayiba N.K., Tshibangu Kabamba E., Kido Y., Speybroeck N. (2023). Malaria drug resistance landscape in the Democratic Republic of the Congo: A spatial mapping systematic review of molecular surveillance surveys. Trop. Med. Health.

[54] Mordeniz C. (2019). Introductory Chapter. Traditional and Complementary Medicine.

[55] Ampomah I.G., Devine S., Ampomah G.A., Emeto T.I. (2024). The ‘STRICT’ framework for promoting effective malaria control in Ghana. Malar. J..

[56] Mordeniz C. (2019). Traditional and Complementary Medicine.

[57] Dauenhauer P., Shields M., Sloughter J.M., Stewart A., Lacrampe C., Magness E., Ochavillo J., Limfueco J., Mendoza A. (2018). Improving Shoestring Surveys for Off-Grid Humanitarian Power Projects: Kilowatts for Humanity and KoboCollect. Proceedings of the 2018 IEEE Global Humanitarian Technology Conference (GHTC).

[58] Vandenbroucke J.P., Elm Evon Altman D.G., Gøtzsche P.C., Mulrow C.D., Pocock S.J., Poole C., Schlesselman J.J., Egger M., Strobe Initiative (2007). Strengthening the Reporting of Observational Studies in Epidemiology (STROBE): Explanation and Elaboration. PLoS Med..

[59] Cochran G.L., Cochran H.C., Ernst M.E. (2025). Research and scholarly methods: Mitigating information bias. JACCP J. Am. Coll. Clin. Pharm..

[60] Savchenko E., Rosenfeld A. (2024). Authorship conflicts in academia: An international cross-discipline survey. Scientometrics.

[61] Elson W.H., Kawiecki A.B., Donnelly M.A.P., Noriega A.O., Simpson J.K., Syafruddin D., Rozi I.E., Lobo N.F., Barker C.M., Scott T.W. (2022). Use of mobile data collection systems within large-scale epidemiological field trials: Findings and lessons-learned from a vector control trial in Iquitos, Peru. BMC Public Health.

[62] Althubaiti A. (2016). Information bias in health research: Definition, pitfalls, and adjustment methods. J. Multidiscip. Healthc..

[63] Meier-Scherling C.P.G., Watson O.J., Asua V., Ghinai I., Katairo T., Garg S., Conrad M., Rosenthal P.J., Okell L.C., Bailey J.A. (2024). Selection of artemisinin partial resistance Kelch13 mutations in Uganda in 2016–22 was at a rate comparable to that seen previously in South-East Asia. medRxiv.

[64] Baina M.T., Djontu J.C., Ntabi J.D.M., Mapanguy C.C.M., Lissom A., Vouvoungui C.J., Boumpoutou R.K., Mouanga A.M., Nguimbi E., Ntoumi F. (2024). Polymorphisms in the Pfcrt, Pfmdr1, and Pfk13 genes of Plasmodium falciparum isolates from southern Brazzaville, Republic of Congo. Sci. Rep..

[65] Young N.W., Gashema P., Giesbrecht D., Munyaneza T., Maisha F., Mwebembezi F., Budodo R., Leonetti A., Crudale R., Iradukunda V. (2024). High frequency of artemisinin partial resistance mutations in the Great Lakes region revealed through rapid pooled deep sequencing. J. Infect. Dis..

[66] Appiah B., Amponsah I.K., Poudyal A., Mensah M.L.K. (2018). Identifying strengths and weaknesses of the integration of biomedical and herbal medicine units in Ghana using the WHO Health Systems Framework: A qualitative study. BMC Complement. Altern. Med..

[67] Chebii W.K., Muthee J.K., Kiemo K. (2020). The governance of traditional medicine and herbal remedies in the selected local markets of Western Kenya. J. Ethnobiol. Ethnomed..

[68] Onyambu M.O. (2019). A Review of Trends in Herbal Drugs Standardization, Regulation and Integration to the National Healthcare Systems in Kenya and the Globe. Int. J. Pharmacogn. Chin. Med..

[69] Onukansi F.O., Anokwuru C.C., Eneh S.C., Odey G.O., Onah D.O., Sokunbi T., Innocent D.C., Dozie U.W., Umoke P.C., Ojo T.O. (2025). Investing in traditional medicine: Leveraging evidence and innovative research to strengthen the fight against malaria in Nigeria. Malar. J..

[70] Rodríguez-Morales A.J. (2021). Current Topics and Emerging Issues in Malaria Elimination.

[71] Weathers P.J. (2025). Medicinal plants as more sustainable therapeutic solutions: Artemisia annua and Artemisia afra as case studies. Glob. Health Econ. Sustain..

[72] Sendor R., Banek K., Kashamuka M.M., Mvuama N., Bala J.A., Nkalani M., Kihuma G., Atibu J., Thwai K.L., Svec W.M. (2023). Epidemiology of *Plasmodium malariae* and *Plasmodium ovale* spp. in Kinshasa Province, Democratic Republic of Congo. Nat. Commun..

