# Correction: Munyangi wa Nkola et al. Public Health Perspectives on Integrating *Artemisia annua* Tea for Uncomplicated Malaria Treatment: A Cross-Sectional Study of Perceptions and Acceptability Among Healthcare Workers in Kalima District, Maniema, DRC. *Trop. Med. Infect. Dis.* 2026, *11*, 105

**DOI:** 10.3390/tropicalmed11100292

**Published:** 2026-10-15

**Authors:** Jérôme Munyangi wa Nkola, Pierre Akilimali Zalagile, Hendrick Lukuke Mbutshu, Spartacus Kabala Munyemo, Imani Ramazani Bin Eradi, Alioune Camara

**Affiliations:** 1 PhD Program in Public Health, Doctoral School in Life Sciences, Health and Environment, Faculty of Health Sciences and Techniques, Department of Medical Sciences, Gamal Abdel Nasser University of Conakry, Conakry BP 1147, Guinea; 2 Faculty of Medicine, University of Kolwezi, Kolwezi BP 453147, Democratic Republic of the Congo; 3 Department of Nutrition, Kinshasa School of Public Health, University of Kinshasa, Kinshasa BP 11850, Democratic Republic of the Congo; pierretulanefp@gmail.com; 4 Patrick Kayembe Research Center, Kinshasa School of Public Health, University of Kinshasa, Kinshasa BP 11850, Democratic Republic of the Congo; 5 School of Public Health, University of Lubumbashi, Lubumbashi BP 1825, Democratic Republic of the Congo; hendrickmbutshu@gmail.com; 6 Academic General Secretariat, University of Kindu, Kindu BP 122, Maniema, Democratic Republic of the Congo; spartacusk@hotmail.com; 7 Nursing Sciences Research, Laboratory of Education and Health Practices, UFR SMBH, University Paris, Sorbonne Paris Cité, F-93017 Bobigny, France; imaniramazani@gmail.com; 8 Nursing Sciences Section, Higher Institute of Medical Techniques of Kindu, Kindu BP 304, Democratic Republic of the Congo; 9 Department of Public Health-Epidemiology, Faculty of Sciences and Health Techniques, Gamal Abdel Nasser University, Conakry BP 1147, Guinea; aliounec@gmail.com; 10 National Malaria Control Programme, Conakry BP 6339, Guinea

## Error in Table

In the original publication [1], there was a mistake in Table 4. Specifically, the table header and specific variable labels contained minor alignment issues, and the dichotomous encoding rule for the dependent variable was omitted from the explanatory caption. The corrected Table 4 appears below.

## Error in Data Availability Statement, Funding and Conflicts of Interest

In the original publication [1], the repository hyperlinks for Data S1 and Supporting Information Files in the Data Availability Statement were broken, rendering the file temporarily inaccessible to public readers. The updated Data Availability Statement is as follows:

**Data Availability Statement:** All relevant data are within the manuscript and its Supplementary Materials. The raw, anonymized dataset used for the statistical analysis (Cochran formula-based sample of N = 337) is provided as Data S1. The survey instrument used for data collection in the Kalima Health Zone is included as Supplementary S1. Any additional inquiries can be directed to the corresponding author or the ethics committee of the Research Ethics Committee of the Institut Supérieur de Techniques Médicales de Kindu.

In the original publication [1], the Funding and Conflicts of Interest Statements did not provide all information relevant to the funding sources and potential conflicts of interest. The updated Funding and Conflicts of Interest Statements appear as follows:

**Funding:** This research received no external funding. The fieldwork, data collection, and quantitative inquiry conducted in the Kalima Health Zone were entirely supported by the personal resources of the lead author. Affiliated institutions provided institutional support in the form of access to libraries and academic infrastructure. No commercial sponsors or non-profit organizations were involved in the study design, data analysis, or the decision to publish.
**Conflicts of Interest:** Between 2015 and 2021, Dr. Jerome Munyangi wa Nkola served as an unpaid medical volunteer and clinical investigator for associations, notably the Munyangi Foundation (FOMU), a non-profit organization based in the Democratic Republic of the Congo dedicated to clinical research and the documentation of local botanical resources. Dr. Munyangi wa Nkola formally resigned from all operational and consultative roles within these organizations in 2021. This termination of activity occurred prior to his official admission to the doctoral program in 2024 and before the conception of the specific research protocol from which the present study is derived. The remaining authors declare no other financial, institutional, or personal competing interests.

The authors state that the scientific conclusions are unaffected. This correction was approved by the Academic Editor. The original publication has also been updated.

## Figures and Tables

**Table 4:** Multivariable logistic regression Determinants of clinical acceptability (N = 337). Dependent variable: willingness to prescribe or recommend the herbal tea if validated (Yes = 1; No+Don’t know = 0).

Predictor	Adjusted OR	95% CI	Sig. (*p*)
Constant	0.333	[0.074–1.439]	0.1445
Sex: Female (ref. Male)	0.545	[0.253–1.184]	0.1210
Area: Rural (ref. Urban)	0.608	[0.245–1.409]	0.2615
Perceived efficacy: Yes (ref. No)	26.490	[11.331–68.315]	<0.001 ***
Perceived political involvement	1.557	[1.022–2.394]	0.0406 *
Barrier: Administrative constraints	0.293	[0.113–0.739]	0.0099 **
Barrier: Concerns about resistance	0.803	[0.251–2.748]	0.7180
Barrier: Safety concerns	0.243	[0.082–0.730]	0.0108 *
Barrier: Rejection by patients	0.205	[0.060–0.722]	0.0117 *

* *p* < 0.05; ** *p* < 0.01; *** *p* < 0.001. ref. = reference category.
