# Supplementary material for: Public Health Perspectives on Integrating Artemisia annua Tea for Uncomplicated Malaria Treatment: A Cross-Sectional Study of Perceptions and Acceptability Among Healthcare Workers in Kalima District, Maniema, DRC
Source: Trop Med Infect Dis. 2026 Apr 17;11(4):105. doi: 10.3390/tropicalmed11040105 (PMC13119536; doi:10.3390/tropicalmed11040105)
Supplement: Supplementary file 1 [file tropicalmed-11-00105-s001.zip › tropicalmed-4221592-supplementary.pdf]

English Translation of the Questionnaire

Title: Survey Questionnaire for Healthcare Professionals regarding the Integration of *Artemisia annua* Tea

Section A: Knowledge and Perceived Efficacy

- **Q1:** Have you observed resistance or opposition to *Artemisia annua* tea from certain groups (patients, colleagues, authorities)? (Yes/No/Don't know)
- **Q2:** If yes, what is the primary origin of this mistrust? (Scientific skepticism, Preference for conventional treatments, Fear of side effects, Cultural/Traditional issues, Others)

- **Q3:** In your opinion, do healthcare workers have sufficient training and information to advise on *Artemisia annua* tea? (Yes/No/Partially)

- **Q4:** What type of training would be useful? (Mode of action/efficacy, Preparation and dosage, Management of side effects, Communication)

- **Q5:** How would you rate the general knowledge level among patients regarding *Artemisia annua*?

Section B: Benefits, Risks, and Barriers

- **Q6:** What are the perceived advantages of this treatment?

- **Q7:** What are the limits or risks associated with the tea?

- **Q8:** Have you already recommended the use of *Artemisia annua* tea to your patients? (Yes/No)

Title: Survey Questionnaire for Healthcare Professionals regarding the Integration of *Artemisia annua* Tea

- **Q9:** In what context would you recommend it?
- **Q10:** What are the barriers to prescription or recommendation?

Section C: Integration and Public Health Strategy

- **Q11:** Would you like to receive specific training on *Artemisia annua* tea? (Yes/No)
- **Q12:** What support materials would be useful?
- **Q13:** Overall, what is your opinion on integrating *Artemisia annua* tea into uncomplicated malaria management?
- **Q14:** How important is political and health authority involvement?
- **Q15:** Is the provision of clear, evidence-based information a key lever for adoption?

Checklist S1: STROBE Statement for Cross-Sectional Studies

Cocher	Item No.	Recommendation	Section in Manuscript
☐	1	(a) Indicate the study’s design in the title or abstract. (b) Provide an informative summary in the abstract.	Title and ABSTRACT
☐	2	Explain the scientific background and rationale for the investigation.	1.1 to 1.4
☐	3	State specific objectives, including any prespecified hypotheses.	1.5
☐	4	Present key elements of study design early in the paper.	3.1
☐	5	Describe the setting, locations, and relevant dates.	3.1 and 3.3
☐	6	Give eligibility criteria, sources, and methods of selection.	3.2
☐	7	Clearly define all outcomes, exposures, predictors, and confounders.	3.4
☐	8	Give sources of data and details of methods of assessment.	3.3
☐	9	Describe any efforts to address potential sources of bias.	3.6
☐	10	Explain how the study size was arrived at.	3.2
☐	11	Explain how quantitative variables were handled.	3.4 and 3.5
☐	12	Describe all statistical methods (adjusted for confounding, etc.).	3.5
☐	13	Report numbers of individuals at each stage of study.	3.2 and 4.1
☐	14	Give characteristics of study participants and missing data.	4.1
☐	15	Report numbers of outcome events or summary measures.	4.2 and 4.4

Cocher	Item No.	Recommendation	Section in Manuscript
☐	16	Give unadjusted and adjusted estimates with 95% CI.	4.4
☐	17	Report other analyses done (subgroups, barriers, etc.).	4.3
☐	18	Summarise key results with reference to study objectives.	5.1
☐	19	Discuss limitations, taking into account potential bias.	5.5
☐	20	Give a cautious overall interpretation of results.	5.1 to 5.4 and 5.6 to 5.8
☐	21	Discuss the generalisability (external validity) of results.	5.5
☐	22	Give the source of funding and the role of the funders.	Acknowledgements / Conflicts of Interest

Checklist S1 Legend

Checklist S1. STROBE Statement Checklist.

This table presents the completed checklist for cross-sectional studies, in accordance with the *STrengthening the Reporting of OBservational studies in Epidemiology* initiative (Elm et al., 2007). It certifies that the 22 essential items required for the rigorous reporting of observational research have been addressed throughout the manuscript (sections 1.1 to 5.8), thereby ensuring methodological transparency and the reproducibility of findings regarding the acceptability of *Artemisia annua* infusion among healthcare workers in the Maniema province (Vandenbroucke et al., 2007). This approach includes comprehensive details on the study design (utilizing Cochran's formula for sample size determination), electronic data collection via the **KoboCollect** mobile application, and advanced statistical analyses using multivariate logistic regression (Lakshminarasimhappa, 2021; Ryan, 2013).

VERIFICATION OF CONFORMITY WITH THE RESULTS

Table S1. Verification of conformity between survey items and manuscript results.

Questionnaire item (Supplementary S1)	Reported result in manuscript	Statistical value/finding
Q8: Global opinion	Overall clinical acceptability	81.0% (n = 273)
Q6: Training desire	Training requirements	91.4% (n = 308)
Q2: Origin of mistrust	Scientific skepticism	39.8% (n = 134)
Q5: Barriers	Lack of clinical evidence	49.0% (n = 165)
Q9: Political involvement	Determinant of acceptability	p = 0.021
Q5: Resistance risks	Awareness of resistance	14.2% (n = 48)

Conformity Statement

The clinical results reported in the manuscript are strictly derived from the raw data collected through the survey. The primary endpoint overall clinical acceptability corresponds to the aggregated "Very Favorable" and "Favorable" responses to item **Q8** of the survey Secondary endpoints, including identified barriers to prescription and specific training requirements, map directly to items **Q5** and **Q6** respectively.

This precise alignment ensures that the socio-professional reality described in the discussion notably the "policy-practice gap" is backed by quantifiable and reproducible field data from the Kalima health zone This verification confirms the integrity of the dataset used for the multivariate logistic regression analysis.

ENSEIGNEMENT SUPERIEUR ET UNIVERSITAIRE

Institut Supérieur de Techniques Médicales de Kindu « ISTM-KINDU »

Comité d'Ethique de Recherche de l'ISTM-KINDU

« C.E.R.I »

Avis éthique de recherche n° réf : 035/ISTM-KD/C.E.R. I/PRESI/IRBE/2025 du 21 / 08 /2025

A Monsieur Docteur MUNYANGI WA N'KOLA Jérôme,
Investigateur principal et doctorant
à l'Université Gamal Abdel Nassser de CONAKRY.
à Kinshasa.

Concerne : Approbation éthique d'un protocole de recherche doctorale

Monsieur l'Investigateur Principal,

Nous avons le plaisir de vous informer que le Comité d'Éthique de Recherche de l'ISTM-KINDU a minutieusement examiné votre protocole de recherche intitulé « **Perception et Acceptabilité de la tisane d'Artemisia annua dans le traitement du paludisme simple dans la Zone de Santé de Kalima, au Maniema en République Démocratique du Congo** », soumis le 29 Juillet 2025.

Après une évaluation approfondie des aspects éthiques conformément aux normes et directives internationales régissant la recherche en santé impliquant des participants humains, le comité d'éthique de recherche de l'ISTM-KINDU vous accorde son approbation éthique pour cette recherche doctorale. Celle-ci sera réalisée dans la zone de santé de KALIMA, dans la province du Maniema, en République Démocratique du Congo, **du 1er septembre au 1er novembre 2025.**

De plus, les membres du C.E.R.I vous rappellent l'importance de respecter rigoureusement toutes les considérations éthiques associées à la recherche en santé impliquant les sujets humains, telles qu'elles sont décrites dans votre protocole. Ces considérations concernent toutes les étapes de la réalisation de l'étude, y compris la collecte, l'analyse et la gestion des données, jusqu'à la publication des résultats.

Veuillez agréer, **Monsieur l'Investigateur Principal**, l'expression de notre parfaite considération.

COMITE D'ETHIQUE DE RECHERCHE DE L'ISTM-KINDU * C.E.R.I. *

Le président du C.E.R.I

Prof. IMANI RAMAZANI Bin Eradi, M.Sc, MPH, PhD.

Galaxy Z Flip3 5G

Comité d'Ethique de Recherche de l'ISTM-Kindu « C.E.R.I »
Adresse :N° 09, Avenue de L'ISTM, Commune de KASUKU , Kindu/Maniema/RD-Congo
Mail : comethiqueistmkindu@gmail.com, Tél : +243 81 14 89 176

ENSEIGNEMENT SUPERIEUR ET UNIVERSITAIRE

Institut Supérieur de Techniques Médicales de Kindu « ISTM-KINDU »

Comité d'Ethique de Recherche de l'ISTM-KINDU

« C.E.R.I »

Avis éthique de recherche n° réf : 035/ISTM-KD/C.E.R. I/PRESI/IRBE/2025 du 21 / 08 /2025

A Monsieur Docteur MUNYANGI WA N'KOLA Jérôme,
Investigateur principal et doctorant
à l'Université Gamal Abdel Nassser de CONAKRY.
<u>à Kinshasa.</u>

<u>Concerne</u> : Approbation éthique d'un protocole de recherche doctorale

Monsieur l'Investigateur Principal,

Nous avons le plaisir de vous informer que le Comité d'Éthique de Recherche de l'ISTM-KINDU a minutieusement examiné votre protocole de recherche intitulé « **Perception et Acceptabilité de la tisane d'Artemisia annua dans le traitement du paludisme simple dans la Zone de Santé de Kalima, au Maniema en République Démocratique du Congo** », soumis le 29 Juillet 2025.

Après une évaluation approfondie des aspects éthiques conformément aux normes et directives internationales régissant la recherche en santé impliquant des participants humains, le comité d'éthique de recherche de l'ISTM-KINDU vous accorde son approbation éthique pour cette recherche doctorale. Celle-ci sera réalisée dans la zone de santé de KALIMA, dans la province du Maniema, en République Démocratique du Congo, **du 1er septembre au 1er novembre 2025.**

De plus, les membres du C.E.R.I vous rappellent l'importance de respecter rigoureusement toutes les considérations éthiques associées à la recherche en santé impliquant les sujets humains, telles qu'elles sont décrites dans votre protocole. Ces considérations concernent toutes les étapes de la réalisation de l'étude, y compris la collecte, l'analyse et la gestion des données, jusqu'à la publication des résultats.

Veuillez agréer, **Monsieur l'Investigateur Principal**, l'expression de notre parfaite considération.

Le président du C.E.R.I

Prof. IMANI RAMAZANI Bin Eradi, M.Sc, MPH, PhD.

Galaxy Z Flip3 5G

C[illegible] d'[illegible]thique de Recherche de l'ISTM-Kindu « C.E.R.I »
Adresse :N° 09, Avenue de L'ISTM, Commune de KASUKU , Kindu/Maniema/RD-Congo
Mail : comethiqueistmkindu@gmail.com, Tél : +243 81 14 89 176

Institute of Medical Techniques of Kindu ex ISTM-KINDU

Ethics Committee of Research of the ISTM-KINDU

C.E.R.I

Ethical research notice n° **ref: 035/ISTM-KD/C.F.R. I/PRES!/IRBE/2025 of 21/08/2025**

To Mr. Doctor MUNYANGI WA N'KOLA Jerome, Principal Investigator and PhD Student at the University Gamal Abdel Nasser of CONAKRY.

Subject: Ethical approval of a doctoral research protocol

Mr. Principal Investigator,

We have the pleasure to inform you that the Ethics Committee of Research of the ISTM KINDU has meticulously examined your research protocol entitled "**Perception and Acceptability of Artemisia annua tea in the treatment of uncomplicated malaria in the Health Zone of Kalima, in Maniema in the Democratic Republic of Congo,**" submitted on July 29, 2025.

After a thorough evaluation of the ethical aspects in accordance with the international standards and guidelines governing health research involving human participants, the ethics committee of research of the ISTM-KINDU grants you its ethical approval for this doctoral research. This will be carried out in the health zone of KALIMA, in the province of Maniema, in the Democratic Republic of Congo, from September 1 to November 1, 2025.

Furthermore, the members of the C.E.R.I remind you of the importance of strictly adhering to all ethical considerations associated with health research involving human subjects, as described in your protocol. These considerations concern all stages of the study's implementation, including data collection, analysis, and management, up to the publication of results.

Please accept, Mr. Principal Investigator, the expression of our perfect consideration.

The President of the C.E.R.I

IMANI RAMAZANl Bin Eradi, M.$c, MPH, PhD.

Research Department of the ISTM-KINDU

C.E.R.I

Address: N° 9, Avenue de l’ISTM, Commune of KASUKU, Kindu, Maniema/80, Congo

Email: comethiqueistmkindu@gmail.com

Phone: +243 81 1489176.
