# Supplementary material for: Public Health Perspectives on Integrating Artemisia annua Tea for Uncomplicated Malaria Treatment: A Cross-Sectional Study of Perceptions and Acceptability Among Healthcare Workers in Kalima District, Maniema, DRC
Source: Trop Med Infect Dis. 2026 Apr 17;11(4):105. doi: 10.3390/tropicalmed11040105 (PMC13119536; doi:10.3390/tropicalmed11040105)
Supplement: Supplementary file 1 [file tropicalmed-11-00105-s001.zip › tropicalmed-4221592-list of supplementary.pdf]

Data S1. Anonymized raw dataset.

This Excel file contains the coded responses from the 337 healthcare providers surveyed in the Kalima health zone. It includes socio-demographic variables, perceptions of efficacy, and reported clinical practices.

Supplementary S1. Survey Instrument.

The 15-item questionnaire used for data collection via the KoboCollect platform. It includes sections on clinical knowledge, barriers to recommendation, and opinions on integration into formal malaria control programs.

Supplementary S2. Verification of Conformity Table.

A detailed mapping document demonstrating the direct correspondence between the raw survey items (S1 Appendix) and the statistical results (Tables 3 and 4) presented in this manuscript .

Supplementary S3. Ethical Approval Certificate.

Official authorization document from the **Comité d'Éthique de Recherche de l'ISTM-KINDU**, reference 035/ISTM-KD/C.E.R. I/PRESI/IRBE/2025This certificate ensures that all procedures adhered to international ethical standards for research involving human subjects. **Supplementary S3 (a). Original Ethical Approval. Supplementary S3 (b). Ethical Approval Translation.**

Checklist S1. STROBE Statement Checklist.

The completed checklist for cross-sectional studies, ensuring that all 22 items required by the STROBE initiative have been addressed in the manuscript for transparency and reporting quality . This includes detailed explanations of how each STROBE item was considered and implemented during the study design, execution, and reporting phases, thereby enhancing the methodological rigor and reproducibility of our findings.
